# PACO: Python-Based Atmospheric Correction

**DOI:** 10.3390/s20051428

**Published:** 2020-03-05

**Authors:** Raquel de los Reyes, Maximilian Langheinrich, Peter Schwind, Rudolf Richter, Bringfried Pflug, Martin Bachmann, Rupert Müller, Emiliano Carmona, Viktoria Zekoll, Peter Reinartz

**Affiliations:** 1 German Aerospace Center (DLR), Earth Observation Center, Remote Sensing Technology Institute, Photogrammetry and Image Analysis, Oberpfaffenhofen, 82234 Wessling, Germany; Maximilian.Langheinrich@dlr.de (M.L.); Peter.Schwind@dlr.de (P.S.); Rudolf.Richter@dlr.de (R.R.); Rupert.Mueller@dlr.de (R.M.); Emiliano.Carmona@dlr.de (E.C.); Viktoria.Zekoll@dlr.de (V.Z.); Peter.Reinartz@dlr.de (P.R.); 2 German Aerospace Center (DLR), Earth Observation Center, Remote Sensing Technology Institute, Photogrammetry and Image Analysis, 12489 Berlin, Germany; Bringfried.Pflug@dlr.de; 3 German Aerospace Center (DLR), Earth Observation Center, Remote Sensing Data Center, Oberpfaffenhofen, 82234 Wessling, Germany; Martin.Bachmann@dlr.de

**Keywords:** atmospheric correction, remote sensing, Sentinel-2, Landsat-8, DESIS, aerosol optical thickness, water vapor, surface reflectance

## Abstract

The atmospheric correction of satellite images based on radiative transfer calculations is a prerequisite for many remote sensing applications. The software package ATCOR, developed at the German Aerospace Center (DLR), is a versatile atmospheric correction software, capable of processing data acquired by many different optical satellite sensors. Based on this well established algorithm, a new Python-based atmospheric correction software has been developed to generate L2A products of Sentinel-2, Landsat-8, and of new space-based hyperspectral sensors such as DESIS (DLR Earth Sensing Imaging Spectrometer) and EnMAP (Environmental Mapping and Analysis Program). This paper outlines the underlying algorithms of PACO, and presents the validation results by comparing L2A products generated from Sentinel-2 L1C images with in situ (AERONET and RadCalNet) data within VNIR-SWIR spectral wavelengths range.

## 1. Introduction

Earth remote sensing data are based on the terrestrial reflection of the incident solar radiation. This radiation is especially affected by different absorption and scattering processes caused by diverse atmospheric constituents on its way down to the Earth surface and up towards the Earth observation sensors (airborne or in orbit). The spatial and temporal variation in composition and properties of some of such constituents makes the compensation of atmospheric effects an important step in the remote sensing applications to retrieve consistent surface properties [1,2,3]. A recent review of atmospheric correction approaches for imaging spectrometers can be found in [4], which includes the so-called quick atmospheric correction (QUAC) [5], the TAFKAA [6] and the ACORN [7] approaches, the HATCH model [8], as well as ATCOR [9]. For multispectral sensors, operational approaches include approaches for MODIS [10] as well as the several developed for Sentinel-2 [11,12,13,14]. The atmospheric correction is particularly important when comparing remote sensing data (surface reflectance) from different sensors in-orbit under realistic atmospheric conditions [15,16,17]. Within this paper, the term “surface reflectance” is used for simplicity, while, strictly speaking, the hemispherical-directional reflectance factor (HDRF) is retrieved.

To retrieve the Bottom-Of-Atmosphere (BOA) surface reflectance from the TOA (Top-Of-Atmosphere) remote sensing radiance, the influence of different atmospheric parameters must be corrected (e.g., Aerosol Optical Thickness (AOT), water vapor (WV) content, etc...) through a set of different atmospheric simulations and algorithms to analyze the remote sensing data.

PACO software package is designed as a Python library version of the IDL code ATCOR [9], following the same algorithms as in its IDL version (summarized in Section 2). As its predecessor ATCOR, PACO is developed as a multi-sensor atmospheric correction software package, easily portable and embeddable into any processing chain. Therefore, PACO is implemented as the current atmospheric correction of the L2A processor in the Ground Segment of DESIS [18] and EnMAP [19] hyperspectral sensors.

Its portability and its multi-sensor support make it possible to apply the same atmospheric correction to different sensors. The application of the same algorithms and the input data (including same radiative transfer functions) to the data of different sensors reduces the uncertainty between the atmospherically corrected output products of the sensors. A PACO software package currently processes both multi-spectral (Sentinel-2 and Landsat-8) and hyperspectral sensors (DESIS and EnMAP) and additional sensors are planned to be added in the future. For all the supported sensors, the same MODTRAN simulation of the atmosphere at high spectral resolution (Δλ∼0.4 nm) is used to extract the sensor specific radiative transfer functions used during the atmospheric correction. The algorithms applied in the atmospheric correction depend on each sensor spectral characterization (e.g., VNIR versus VNIR-SWIR sensors).

PACO also profits from the large amount of API (Application-Programmming-Interface) in Python in order to process additional information from external sources. Further details will be discussed in Section 3.

Currently, PACO also processes additional information regarding the remote sensing acquisition, as, for example, the LST (Land Surface Temperature) to determine the season, the Ozone column, the site aerosols optical thickness, water vapor, and dead pixel masks. If available, the dead pixel mask is taken into account to select corresponding sensor bands for the analysis and the exclusion of dead pixels from the atmospheric correction algorithms.

This paper presents the software characteristics and its performance validating the atmospheric (see Section 4.1.1) and surface reflectance (see Section 4.1.3) L2A products, obtained with PACO for the multi-spectral sensors Sentinel-2 (A and B) and Landsat-8. In the last section, we compare PACO results between multi-spectral (Sentinel-2) and hyper-spectral (DESIS) L2A results.

## 2. Algorithm Overview

The algorithms implemented in this software are inherited from ATCOR [9,20,21,22,23,24].

As a first step, Look-Up-Tables (LUTs) with radiative transfer (RT) functions have been computed using MODTRAN 5.4.0 [25] for both mid-latitude summer and winter seasons. The simulated radiative transfer functions are transformed to sensor radiative transfer LUTs, convolving them with the sensor spectral response function per band. The same spectral response functions are used to calculate the solar irradiance for the specific sensor using different solar models (e.g., Fontenla [26], Thuillier2003 [27], etc.). The sensor LUTs, containing the simulated radiative transfer functions, are binned in several parameters according to the observation conditions (scene and atmosphere) and the sensor characterization:

- Observation:
  - sun zenith angle (0${}^{\circ }$–70${}^{\circ }$)
  - observation off-nadir angle (0${}^{\circ }$–40${}^{\circ }$)
  - sun-sensor relative azimuth angle (0${}^{\circ }$–180${}^{\circ }$)
  - ground elevation (through the digital elevation model, DEM, if provided) (0–4 km)
- Atmosphere:
  - visibility (or aerosol optical thickness) (5–120 km)
  - water vapor (summer: [0.4–5] cm and winter: [0.2–1.1] cm)
  - aerosol model (rural/continental, urban, maritime and desert)
- Sensor: band central wavelength (same for all pixels across-track). The variation of the band central wavelength across-track, often referred to as “spectral smile”, can be included during the processing adding a set of polynomial coefficients into a sensor file.

The observation and sensor parameters are known a priori, while the atmospheric ones must be retrieved from the EO (Earth Observation) input data itself or assumed during the first steps of the atmospheric correction.

**Figure 1:**
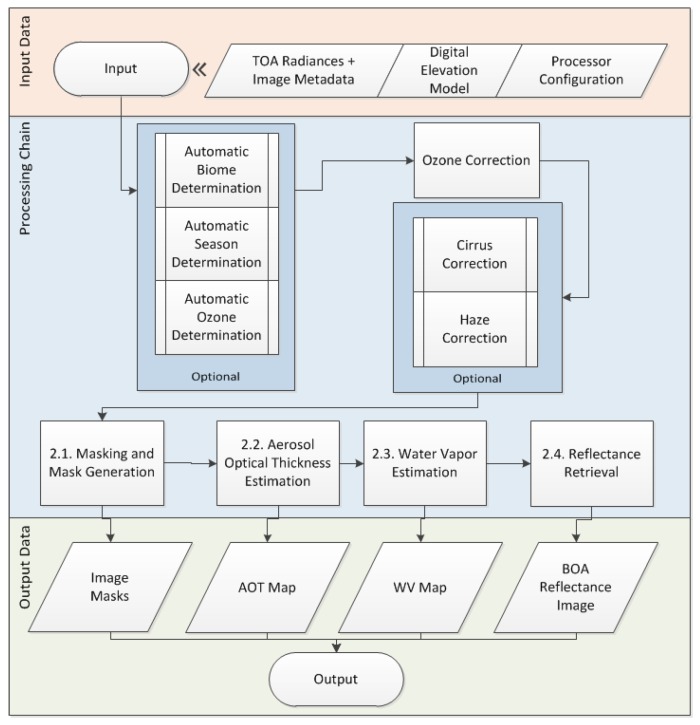
PACO algorithm workflow. The upper and bottom rows contain the input and output products, respectively. In the central row (blue box), the workflow of the algorithms described in Section 2 is displayed. The output products are explained in Section 3, and all of them might be combined in a final Output product if required by the mission.

The first steps of the processor make it possible to determine the ozone column value and the season (summer or winter) from an external source (e.g., MODIS Aqua/Terra products [28,29]), used for the atmospheric correction (see Figure 1). The ozone column value can be also extracted from the input metadata and is used to correct the radiance from the ozone absorption [23]. After the ozone correction, it is possible to remove the haze and/or cirrus present in the scene if specified through the command line.

The next step of the processor classifies the pixels among a pre-defined set of classes. Each class of pixels is considered differently amongst the rest of the atmospheric correction steps. Clear land pixels are classified into non-reference and reference pixels. Reference pixels are Dark Dense Vegetation (DDV), pixels used to determine the atmosphere visibility and the Aerosol Optical Thickness of the scene at 550 nm (Section 2.2). If the sensor has bands at water vapor absorption wavelengths (typically around 820, 940, and 1130 nm), the water vapor column is determined (Section 2.3); otherwise, a default value based on the season is assumed.

Once all the LUTs bins have been determined, the Bottom-Of-Atmosphere reflectance per pixel is calculated (Section 2.4).

### 2.1. Pre-Classification

The classification criteria is performed in the same way as in the ATCOR software [9], using the apparent surface reflectance ($\rho^{*}$) of several bands (multi-spectral (MS) method), plus terrain information and relative sensor and sun positions, with:

(1) $$\rho^{*}=\frac{\pi \cdot d^{2}}{E\cos\theta }\left(c0+c1*DN\right)$$

where DN are the digital counts collected by the sensor, E is the direct solar flux, d is the sun-Earth distance, θ is the sun zenith angle, and c0 and c1 are the offset and band scaling factors, respectively.

PACO produces the same list of masks as ATCOR v9.1 [30], which are saved with its corresponding map:

- Background (0): pixels flagged as background (’background’ data value or values set to zero).
- Shadows (1): shadows identified using spectral information. This mask might include part of the topographic shadows.
- Thin (2), medium (3), and thick (4) cirrus over water: different cirrus thicknesses (based on spectral information) over pixels previously flagged as water. These flagged pixels are excluded from the water mask (17).
- Land (5): include all the pixels not classified by the criteria of all other masks in this list, which in general is equivalent to clear land pixels (e.g., no clouds, cirrus, or haze).
- Saturated (6): pixels likely influenced by sensor saturation.
- Snow/ice (7): pixels classified as snow and snow-ice.
- Thin (8), medium (9) and thick (10) cirrus over land: same spectral classification as in masks 2, 3, and 4 applied over pixels previously classified as land.
- Haze over land (11) and over water (13): previously classified land and water pixels with presence of haze.
- Clouds over land (15): clouds detected through spectral thresholds. If no external water mask is provided, the classification include land and water clouds (mask 16).
- Clouds over water (16): clouds detected over water pixels, which are identified through an external land/water mask.
- Water (17): water pixels under clean atmospheric conditions (e.g., no clouds, cirrus or haze detected in the atmosphere).
- Cirrus cloud (18) and thick cirrus cloud (19): pixels classified as cirrus with a thickness so that no land/water distinction below them can be performed. They are selected using higher thresholds than for masks 2, 3, 4, 8, 9, and 10.
- Bright (20): high apparent reflectance pixels, not classified as cloud or haze.
- Topographic shadows (21): shadows identified through the sun and sensor positions if a Digital Elevation Model (DEM) is provided. It uses a ray tracing algorithm and it includes cast shadow regions.

### 2.2. Aerosol Optical Thickness

The algorithm to derive the AOT is based on the identification of DDV pixels and the negligible scattering and absorption effects of aerosols in the infra-red wavelength range. The visibility is empirically retrieved from the apparent reflectance of the previously identified DDV pixels at VNIR or SWIR wavelengths. For sensors with bands in the SWIR (around 2.2 μm), a similar approach as for MODIS [31] is used. For sensors with only available bands in the VNIR range, the empirical approach uses the red and NIR apparent reflectance of the DDV pixels [20].

The mean visibility of the DDV pixels is considered for the rest of the scene and smoothed over a 3 km low pass filter. For those scenes where there are less than 2% of DDV pixels, a default value of 23 km is assumed as the mean scene visibility. The final AOT is retrieved scaling the obtained visibility per pixels by the terrain elevation (if available) or the mean scene altitude. As a last step, the path radiance in the blue and red bands is scaled according to the reflectance relations in those channels of Dark-Dense Vegetation [31].

### 2.3. Water Vapor

The water vapor column map (in cm) is calculated per-pixel with the Atmospheric Pre-corrected Differential Absorption (APDA) algorithm [32] using the water absorption region at 820 nm, 940 nm, and/or 1130 nm. The absorption band to be used depends on the band characterization of each sensor. They can be specified through a configuration file per scene; otherwise, the software configures them automatically according to a predefined wavelength range. Additionally, an option in the processor configuration allows for using one or several bands in the algorithm.

It is possible, through a configuration parameter, to use both absorption regions at 940 and 1130 nm for the water vapor estimation.

For sensors with several bands in any of these absorption regions, as, for example, hyperspectral sensors, a linear regression of several bands is used in order to minimize the individual channel noise.

### 2.4. Bottom-Of-Atmosphere Reflectance

Once the corresponding radiative transfer functions are known per pixel and wavelength, the radiative transfer equation for a homogeneous flat surface under clear sky conditions can be formulated as:

(2) $$L=L_{p}+\left(\tau_{dir}+\tau_{dif}\right)\frac{\rho }{\pi }\frac{\left(E_{dir}\cos\theta_{s}+E_{dif}\right)}{1-\rho \cdot s}$$

where ρ is the Lambertian BOA surface reflectance, L is the at-sensor radiance, $\theta_{s}$ the sun zenith angle, and L${}_{p}$, $\tau_{dir}$, $\tau_{dif}$, E${}_{dir}$, E${}_{dif}$, and *s* are the path radiance, ground-to-sensor transmittance (direct and diffuse), direct and diffuse solar flux on the ground, and spherical albedo of the atmosphere, respectively. The dependence of some parameters with the band wavelength, sun and viewing geometry, and atmospheric parameters has been omitted from the equation for simplicity but are implicit in the RT LUTs.

As described in [9], the surface reflectance is retrieved by correcting the radiance image, taking into account the adjacency effect [33,34], caused by photons from the surrounding area that are scattered into the sensor’s line-of-sight.

This approach is applied when no digital elevation model is provided as part of the input parameters, and therefore a flat-terrain atmospheric correction is performed. If the mean altitude of the scene is known, a flat DEM with the mean scene altitude is created inside the scenario. If the mean altitude is not provided, sea-level is considered as the default scene mean altitude. This flat-terrain atmospheric correction is applied if less than 1% of the scene contains pixels with slopes > 6 degrees, even if the DEM is provided. Otherwise, the rugged-terrain scenario is considered for the atmospheric correction, computing maps of slope and aspect from the DEM provided. This combined atmospheric and topographic correction demands more complex equations, described in more detail in [9]. The rugged-terrain local illumination ($\cos(\theta_{sun})$) per pixel, as considered during the surface reflectance, is saved as a by-product.

## 3. Dependencies and Technical Specifications

The PACO atmospheric correction software is implemented through a modular library utilizing the Python programming language (version 2.7). Besides discarding the dependency of an IDL license, the use of Python brings further advantages that are commonly spread through the scientific community:

- Availability of a multitude of highly developed scientific computing packages on the basis of open source licences (dependencies are described in Section 3).
- Maintenance and updating of third party modules is driven forward by a large community, enabling fast and in many cases highly adjusted solutions to difficulties concerning software development.
- The relatively simple and easy to read syntax of the Python language speeds up prototyping, implementation, debugging, and collaboration.
- Inter-operability: interaction with most other languages and platforms through 3rd party modules.

Apart from these general motivating factors concerning the development language of PACO, several design choices were made during development that address the architecture of the overall framework:

- Different procedural sections of the overall algorithm are organized in individual modules (i.e., masking/classification of pixels, water vapor correction, AOT estimation, etc.). This enables easy extension of established modules with new algorithms as well as modular design of sensor specific processing pipelines.
- Sensor specific processing is organized as individual scenarios addressing particular sensor characteristics (i.e., multi-resolution data acquisition of Sentinel-2 MSI), while relying on the same baseline of modules concerning the essential atmospheric correction steps.

The current PACO software stable release version for Sentinel-2 and Landsat-8 users is 0.9.1.

It depends on the following external libraries:

- numpy (v1.11.0),
- scipy (v0.17.0),
- gdal (v1.11.3).

The software allows for performing the atmospheric correction both in ortho-rectified TOA radiances (or TOA reflectances) images as well as in sensor geometry. In the latter case, only the size in meters of the square pixel must be provided in the input metadata.

The software uses XDIBIAS libraries (satimport module, developed at DLR) and its data model structure to load each sensor data and metadata. The output can be stored in XDIBIAS format. Alternatively, other common formats can be specified for the output images (e.g., ENVI, TIF, GeoTIFF, etc.). The metadata output is currently stored in JSON format.

### Performance and Output Products

As described in Section 2, PACO produces not only the final BOA surface reflectance products, but also a set of intermediate products that were calculated and used during the atmospheric correction.

In addition, the PACO specific intermediate products (like AOT, water vapor, masks, ...) are saved and tagged according to Table 1.

Description:

- Pre-classification mask (“hcw”): pixel mask of coded 8-bit integer flag, following the criteria and the flagging criteria detailed in Section 2.1.
- Dense Dark Vegetation pixel mask(“ddv”): byte-wise subset mask, where background (0), water (1) and dark-dense vegetation pixels (2) are flagged. The rest of them are flagged as non-reference pixels (3).
- Visibility index map (“visindex”): atmospheric visibility index per pixel. Each index corresponds to a visibility (in km), related with an AOT at 550 nm. The corresponding tables are included within the software.
- Aerosol optical thickness map (“aot”: estimated AOT at 550 nm (dimensionless) per pixel as described in Section 2.2.
- Water vapor column map (“ wv”): water vapor column (in cm) estimated through step (Section 2.3)
- Illumination map (“ ilu”): cosine of local solar incidence angle.
- BOA surface reflectance (“L2A”): final surface reflectance (after BRDF correction) measured in “percent (%) * 100”. The resulting bands are sensor dependent and are detailed in Table 2.

The original spatial dimensions are preserved (number of rows × number of columns) and are the same for all products, therefore only the 3rd dimension changes along the products. The spectral dimensions (“bands”) of the BOA surface reflectance files depends on the sensor, and in the case of Sentinel-2, also on the resolution (see Table 2):

Together with the previously mentioned products, a metadata file with scene, geographic, and atmosphere information is saved in JSON format.

## 4. Validation of PACO Atmospheric Correction

The accuracy of the BOA surface reflectance retrieval depends on the accuracy of different variables used within the atmospheric correction algorithms. The first ones are those affecting the input data itself, as the instrument calibration, the simulation of the radiative transfer functions, the solar irradiance spectra, the ozone column or the DEM. They depend on the accuracy of instruments and algorithms external to PACO, and they are not discussed here. However, other atmospheric parameters like the AOT and WV are estimated inside the atmospheric correction software using the EO data (if they are not explicitly given as input parameters), and they might have a considerable effect on the final results. Therefore, these parameters are validated in this study.

The algorithms that estimate the AOT (τ) at 550 nm in optical remote sensing data are proved to be related to the accuracy in the final retrieval of the BOA surface reflectance [20,31] in blue and green wavelengths through:

(3) $$\Delta \rho =0.1\cdot \Delta \tau_{550}$$

If the apparent reflectance in the SWIR bands is $\rho_{2.2\mu m}$ < 0.1 [31], a surface reflectance absolute difference of Δρ = ± 0.006 at 490 and 660 nm, for dark-dense vegetation pixels, corresponds to an error in the aerosol optical thickness retrieval of ΔAOT${}_{550}$∼± 0.06.

For completeness, the study includes the check of the Water Vapor values with AERONET (AErosol RObotic NETwork) stations [37], following the same approach as mentioned by [38]. According to the results in [38], a relative error between 8 and 10% is expected for Sentinel-2 datasets.

The accuracy (A), precision (P), and uncertainty (U) are calculated for each atmospheric parameter following [39] (see Equation (4)):

(4) $$A=\frac{1}{N}\sum \left(x_{i}-x_{ref}\right)\;;\;P=\sqrt{\frac{1}{N-1}\sum {\left(x_{i}-x_{ref}-A\right)}^{2}}\;;\;U=\sqrt{\frac{1}{N}\sum {\left(x_{i}-x_{ref}\right)}^{2}}$$

where x${}_{i}$ is the parameter calculated from remote sensing data and x${}_{ref}$ is the measurement from AERONET and RadCalNet for atmospheric parameters and surface reflectance validation, respectively. The accuracy (also called trueness) measures the statistical bias while the precision gives an indication of the statistical variability of the estimate. The uncertainty measures the statistical deviation of the estimate of the truth, including the mean bias.

The three statistical values are calculated over a collection of scenes for the atmospheric parameters (one reference value per scene) and over a collection of pixels in the case of the surface reflectance (one reference value for several pixels in a region of interest). They are compared with previous results and between the different sensors currently implemented within PACO.

### 4.1. Atmospheric Parameters: AOT and WV versus AERONET Data

**Figure 2:**
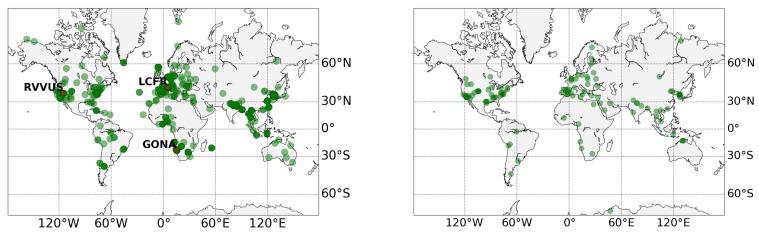
Distribution of the AERONET stations (green circles) used in the Sentinel-2 (**left**) and Landsat-8 (**right**) validation study. The RadCalNet sites used for the Sentinel-2 surface reflectance validation are shown in the left plot (red stars): RailroadValley Playa (RVUS), La Crau (LCFR), and Gobabeb (GONA).

The validation of the atmosphere characterization products, AOT and WV, is done with reliable, independent and well calibrated in situ measurements from the AErosol RObotic NETwork global data set of sun-photometers [37]. This validation study is based on Level 2.0 (quality assured) of AERONET products (version 3). The spatial distribution of the AERONET sites used in this study is shown in Figure 2.

#### 4.1.1. Atmospheric Parameters’ Data Sets

The validation between satellite data and AERONET measurement is performed on multiple scenes that fulfill several overpass criteria (atmosphere-based) that foresee the same atmospheric conditions between reference and remote sensing data sets:

1. Wavelength: the value of the AOT is wavelength dependent, so we must interpolate the measured AERONET AOT data at different wavelengths to its value at 550 nm.
2. Time: the AERONET measurement is interpolated to the satellite overpass time using measurements in the time interval ± 30 min for the interpolation.
3. Space: to ensure we are measuring the same atmospheric path, the satellite measurement is extracted from a 9 × 9 km region of interest (ROI_9) around the AERONET coordinates.

In addition to the atmospheric overpass criteria, each of the atmospheric parameters is evaluated over a subset of scenes:

1. WV:
  (a) Clear land: the WV statistics are computed based on clear land (no clouds, cirrus, water, etc..) pixels contained in the ROI_9, with a minimum of 5% of land pixels.
2. AOT:
  (a) Number of DDV pixels > 5%: in addition to the previously clear land region, the AOT statistics per scene are extracted among the DDV pixels present in the region of interest (ROI_9).

The analysis is performed under these two sets of conditions.

#### 4.1.2. Results on Atmospheric Parameters Retrieval for Multi-Spectral Sensors: Sentinel-2 and Landsat-8

The accuracy (A), precision (P), and uncertainty (U) (Equation (4)) have been calculated for the AOT and WV centered in Sentinel-2 and Landsat-8 scenes. For Landsat-8 only, the AOT validation is performed since the sensor does not contain any band center in moderate water vapor absorption regions. The results are summarized in Table 3.

**Figure 3:**
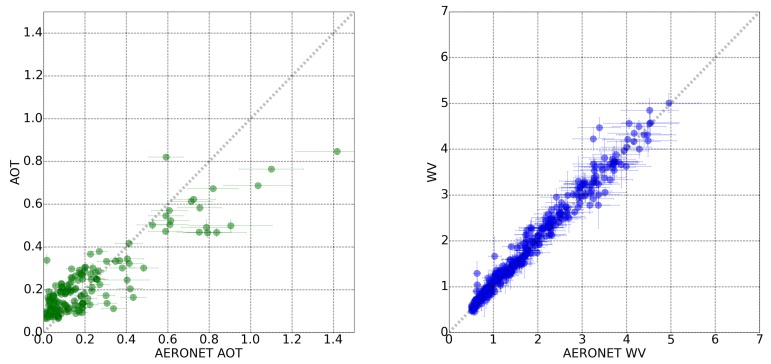
AOT (**left**) and WV (**right**) correlation plots between AERONET measurements and values retrieved by the remote sensing algorithm in a total of 494 Sentinel-2 scenes.

The corresponding correlation plots are shown in Figure 3 for the Sentinel-2 sensor. The error bars on the *x*-axis correspond to the quadratic sum of the AERONET instrumental error (10% of reference value) and the statistical error due to the interpolation in time. The error bars on the *y*-axis are related to the statistical fluctuations in the image.

The linear correlation between the WV calculated and the measured one by AERONET stations (Figure 3, right) has a Pearsons coefficient (r) of 0.99. All the points are distributed along the correlation line (grey dashed line) for the full interval of water vapor values between 0.5 and 5.0 cm. However, a lower linear correlation exists between the AOT measurements and the ones from AERONET (r = 0.89) in the range of 0.006 to 1.4. In Figure 3 (left), a clear underestimation of the AOT for AERONET measurements above ∼ 0.7 is visible. A smaller overestimation of the AOT is seen for very low values of the AOT.

**Figure 4:**
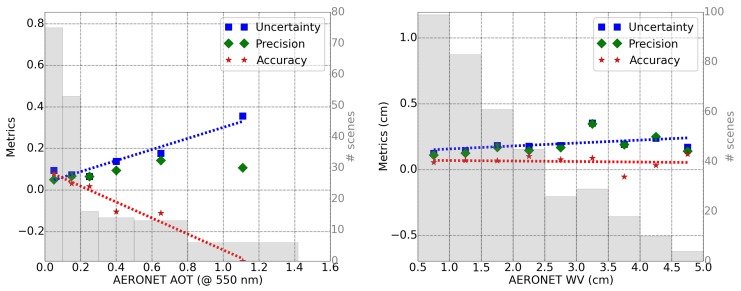
Accuracy (red star), precision (green diamond) and uncertainty (blue square) for calculated AOT (**left**) and WV (**right**) for Sentinel-2 as a function of the reference values (AERONET). The number of scenes included in each bin are shown in the shadow histogram and in the right axis. The accuracy and uncertainty fits are displayed as dotted red and blue lines, respectively.

In order to study possible systematic biases, the accuracy, precision, and uncertainty are calculated as a function of the reference value (AERONET measurements), as performed in [40,41] and displayed in Figure 4. The linear fit to the uncertainty (blue dotted line) and accuracy (red dotted line) are also displayed.

The accuracy represents a mean bias error, which is approximately constant for the water vapor ($A_{WV}=0.004*WV+0.07$, in cm) but dependent on the reference value for the AOT estimation ($A_{AOT}=-0.4*AOT+0.1$, at 550 nm). However, the uncertainty is clearly dominated by the precision of the retrieval method for both WV and AOT. Only for very high values of AOT (>0.8) does the lack of accuracy increase the uncertainty significantly. Therefore, the uncertainty in the retrieval of AOT and WV values is $U_{AOT}\left(550\;\mathrm{nm}\right)=0.29*AOT\left(550\;\mathrm{nm}\right)+0.03$ and $U_{WV}\left(\mathrm{cm}\right)=0.02*WV\left(\mathrm{cm}\right)+0.13$, respectively.

The different statistics from the particular distribution population used in this validation study are presented in Table 3 for Sentinel-2 and Landsat-8 sensors.

In Sentinel-2 and Landsat-8, the results are consistent with the study on Landsat and Rapid Eye scenes performed by [42]. The mean accuracy obtained by [42] was ∼0.04, which is inside a precision of 0.12. As we have seen, in the case of AOT statistics, the distribution of AOT values of our population must be taken into account. Adding scenes with AOT larger than 0.7 to our population, results in an increase of the accuracy value of this population due to lower accuracy for higher AOT values. Calculating the median of our distribution, the AOT uncertainty is ∼ 10% for Sentinel-2. As mentioned before, the water vapor validation is performed for Sentinel-2. Both the uncertainty and accuracy results are consistent with previous studies of the ATCOR performance, where an uncertainty value below 10% is reported [38].

The results from aerosol optical thickness and water vapor for Sentinel-2 are slightly higher than ESA-L2A products (Sentinel-2 L2A core products) (Sentinel-2 products systematically generated at the ground segment since March 2017 [43]). after the update of the Sentinel-2 response functions (the same response functions used in our study). The Sentinel-2 study in [43] reports an accuracy (MBE) = 0.01 and an uncertainty (RMSE) = 0.08, for a distribution with AOT values between 0. and 0.8. Figure 3 shows uncertainty values below 0.08 for all AOT bins between 0.1 and 0.4. From 0.4 to 0.8, the uncertainty increases up to ∼ 0.18.

For the water vapor, the study presented here shows better results in the uncertainty and the accuracy, compared to the values in [43] (RMSE = 0.24 cm and MBE = −0.14 cm).

According to Equation (3), these results foresee an increase of 0.003 in the surface reflectance difference (decrease of accuracy) due to an AOT mean accuracy of 0.03. An error of 10% in the WV estimation would add up to ∼0.7% of uncertainty to the surface reflectance accuracy above 700 nm.

#### 4.1.3. Surface Reflectance: Comparison to RadCalNet Sites Data

The surface reflectance is validated following the same criteria as proposed in [39] for Landsat-8. In this paper, the difference between the retrieved surface reflectance (ρ) per pixel and the reference value falls within the MODIS theoretical uncertainty of |Δρ| ≤ 0.05*$\rho_{ref}$ + 0.005 [39], which corresponds to ∼ 5% relative difference in surface reflectance.

The validation exercise in [39] compares surface reflectance with simulations over AERONET sites, using the atmospheric parameters measured by AERONET station. Since simulated data over AERONET sites are not available, the validation exercise presented in this paper uses the publicly available surface reflectance measurements on the RadCalNet sites [44,45] as reference data. This RadCalNet reference data are compared with PACO BOA surface reflectance retrieved from Sentinel-2 satellite scenes.

These sites provide measurements for a certain extended area, typically flat and approximately Lambertian [45]. Therefore, the BOA reflectance compared in this study does not include the application of any Bidirectional Reflectance Distribution at a surface.

Since these sites are intended for validation of instrument calibration, they only make data available when the atmospheric conditions are stable and clear. For this reason, the effect of large aerosols or water vapor concentration in the retrieved surface reflectance can not be tested with these reference data.

The Baotou RadcalNet site is excluded from this study due to a small validation area (<48 m) [46].

#### 4.1.4. Surface Reflectance Data Sets

To perform a good quality atmospheric correction, the atmospheric parameters must be properly retrieved. In order to minimize the atmospheric effects on the surface reflectance validation results, the validation sites are characterized by bright surfaces, typically arid or deserts, with few or no season dependency. This represents a challenge for atmospheric correction algorithms based on the spectral properties of dark dense vegetation, like PACO.

In this exercise, the overpass criteria spectral-based) must fulfill the following:

1. Wavelength: the original ground truth spectra ($\rho_{RCN}$) are convolved to the sensor RSP functions per band (see Equation (5)) to obtain the sensor-wise RadcalNet spectra ($\rho_{RCN,S2}$)
2. Space: each RadcalNet site has different extended area for validation, corresponding to different surface reflectance homogeneity (see Table 4).
3. Time: surface reflectance is extracted within the same day. The RadCalNet spectra measurements (per band) are linearly interpolated in time using the good quality measurements of the day (typically spaced each 30 min). Scenes without good quality RadCalNet measurements right before/after the remote sensing time are also discarded from the study to avoid possible bad weather conditions (clouds) that might have occurred:
  (5) $$\rho_{RCN,S2}=\frac{\int_{S2_{\lambda }}\rho_{RCN}\cdot SRF_{S2,\lambda }}{\int_{S2_{\lambda }}SRF_{S2,\lambda }}$$

For this study, a total of eight scenes for La Crau and Railroad Valley Playa and 11 for Gobabeb sites have been considered.

#### 4.1.5. Results on BOA Reflectance Retrieval for Multi-Spectral Sensors: Sentinel-2

The same statistics, as for the atmospheric parameters (accuracy (A), precision (P), and uncertainty (U) (see Equation (4))), are used in this last validation exercise. The surface reflectance retrieved for each pixel contained over the delimited area for each RadCalNet site (Table 4) is compared with the RadCalNet BOA reference value.

**Figure 5:**
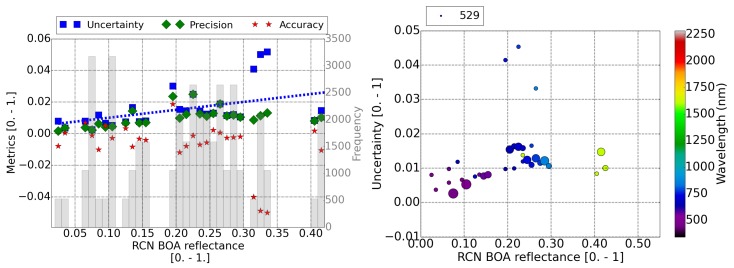
(**Left**) Accuracy (red star), precision (green diamond), and uncertainty (blue square) as a function of the reference value of Sentinel-2 surface reflectance for the La Crau RadCalNet site. The number of pixels included in each bin are shown in the shadow histogram and on the right axis. Uncertainty fit is displayed as a dotted blue line. (**Right**) Uncertainty of the surface reflectance versus the reference value and the band central wavelength. The size of the points scales with the amount of pixels analyzed in each point. The amount of pixels considered for the smaller point is displayed in the legend. In both plots, the swir band (2250 nm), cirrus band (1380 nm), and water absorption bands at 940 nm have been discarded.

**Figure 6:**
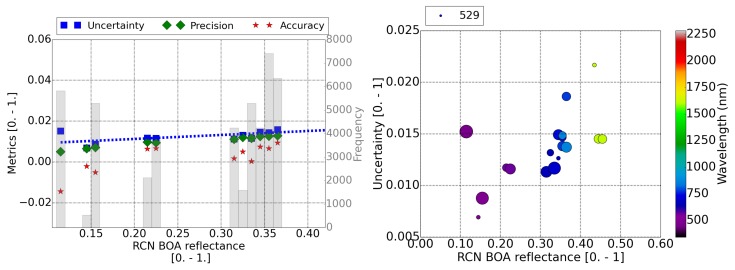
(**Left**) Distribution of the accuracy, precision, and uncertainty of Sentinel-2 surface reflectance as a function of the reference surface reflectance for the Gobabeb RadCalNet site. The number of pixels included in each bin are shown in the shadow histogram and on the right axis. Uncertainty fit is displayed as a dotted blue line. (**Right**) Uncertainty of the surface reflectance versus the reference value and the band central wavelength. The size of the points scales with the amount of pixels analyzed in each point. The amount of pixels considered for the smaller point is displayed in the legend. In both plots, the swir band (2250 nm), cirrus band (1380 nm), and water absorption bands at 940 nm have been discarded.

**Figure 7:**
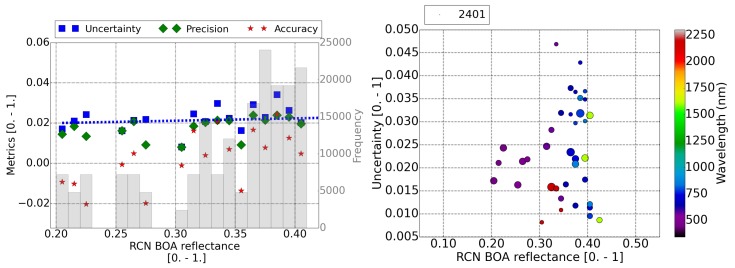
(**Left**) Distribution of the accuracy, precision, and uncertainty of Sentinel-2 surface reflectance as a function of the reference surface reflectance for the Railroad Valley Playa RadCalNet site. The number of pixels included in each bin are shown in the shadow histogram and on the right axis. Uncertainty fit is displayed as the dotted blue line. (**Right**) Uncertainty of the surface reflectance versus the reference value and the band central wavelength. The size of the points scales with the amount of pixels analyzed in each point. The amount of pixels considered for the smaller point is displayed in the legend. In both plots, the swir band (2250 nm), cirrus band (1380 nm), and water absorption bands at 940 nm have been discarded.

Figure 5, Figure 6 and Figure 7 contain the results for the three RadCalNet sites (La Crau, Gobabeb, and Railroad Valley Playa, respectively) under study. The plots on the left display the metrics (A, P and U) of the retrieved reflectance per pixel as a function of the reference surface reflectance (Equation (4)). The fitted function to the calculated uncertainty points, as a function of the surface reflectance, is displayed as a blue dotted line. These plots on the left assume that all the bands with the same surface reflectance contribute equally to the total uncertainty of the surface reflectance bin. This assumption is taken considering that the current uncertainty limits [39] are expressed only as a function of the surface reflectance. This is checked for this validation study with the scatter plots on the right, where the uncertainty is displayed as a function of the surface reflectance and the central wavelength of the band (color scale). The size of the points in the scatter plot are scaled to the amount of pixels analyzed and the amount of pixels for the smaller point is specified in the legend.

La Crau is the only RadCalNet site where, depending on the time of the year, one might be able to identify enough DDV pixels to estimate the AOT of the scene and use it for the atmospheric correction. All the scenes considered in this study have at least 5% of DDV pixels. The linear fit of the uncertainty for La Crau site (blue dotted line in the left in Figure 5), as a function of the reference surface reflectance ($\rho_{ref}$), yield $U_{\rho ,BOA}=0.05*\rho_{ref}+0.005$, in units of surface reflectance between 0. and 1. This result is consistent with MODIS limits [39]. The left plot in Figure 5 shows some reflectance bins at ∼0.30 with larger uncertainties caused by a lack of accuracy of the reflectance of ∼4%. The right plot in Figure 5 shows that the large uncertainty is mainly due to SWIR band.

A possible explanation for the difference in the SWIR band could be the limitation of RadCalNet spectra data up to 2300–2400 nm for some sites. The Sentinel-2 SWIR band centered at 2200 nm has a FWHM of ∼174 nm, so, for these sites, with a limited spectra range, the interpolated RSP misses around 5%–10% of the signal.

This seems to be the case for the La Crau and Gobabeb sites. For these sites, the SWIR band is discarded from the analysis because the extrapolation might yield different results between the convolution of RadCalNet spectrum and the actual sensor measurement for this specific band.

For the considered surface validation area of the Gobabeb site (Figure 6), a variability across site validation area of 3% is expected. The uncertainty results, fitted to a linear function, are $U_{\rho ,BOA}=0.04*\rho_{ref}+0.005$ (blue dotted line in Figure 6, left), which are also consistent with MODIS uncertainty limits. At blue wavelengths (≤500 nm) (Figure 6, right), the overestimation of the AOT causes a lower surface reflectance when compared to RadCalNet reference measurements (mean accuracy ∼−0.02). This is the main cause for the increase in the uncertainty.

The last of our validation sites, Railroad Valley Playa (Figure 7), is also an arid site as Gobabeb. However, the surface reflectance results for this site show a much larger uncertainty of $U_{\rho ,BOA}=0.01*\rho_{ref}+0.018$ than for the Gobabeb site. This uncertainty seems not to be correlated with any band (right plot in Figure 7), and it is above the MODIS limits (>5%), although the site variability (∼1.5%) is the smallest of the three RadCalNet sites. The similar size of the points in the right plot in Figure 7 suggests a rather homogeneous distribution in the number of pixels for the different surface reflectance values. The atmosphere conditions of the Railroad Valley data sets were the same as for Gobabeb. No DDV pixels are found in these two arid sites. Therefore, the AOT value used for the atmospheric correction can be easily a factor 10 larger than measured values, especially for sites where data are only publicly available for clear atmospheric conditions. This could translate to an increase of the uncertainty of ∼3% for some scenes according to Equation (3), since, for clear conditions (AOT∼0.03 measured by RadCalNet), the default visibility of 23 km assumed by the remote sensing algorithm corresponds to AOT ∼ 0.25. For this reason, an additional uncertainty of ∼3% could contribute to the final results for these types of scenes. However, results for both sites are quite different. Further research is currently underway.

For two (Gobabeb and La Crau) of the three validation sites, the precision of the surface reflectance retrieval is within MODIS limits of $0.05*\rho_{ref}+0.005$. However, for the arid site (Gobabeb), this uncertainty has a larger offset (∼0.007) due to the larger accuracy value at blue wavelengths.

## 5. Comparison of BOA Surface Reflectance between Multi-Spectral and Hyper-Spectral Sensors: Sentinel-2 versus DESIS

The latest exercise in our validation study compares the surface reflectance obtained for scenes for different sensors fulfilling the spectral overpass criteria (Section 4.1.3). For this purpose, we use as an example the data acquired by DESIS and Sentinel-2 over one of the RadCalNet sites (Railroad Valley Playa) on 28 June 2019. The validation of DESIS surface reflectance for different RadCalNet sites is fully reported in [18]. Hence, we compare the results versus the corresponding Sentinel-2 scene.

The summary of the data acquisitions of DESIS and Sentinel-2 is detailed in Table 5.

**Figure 8:**
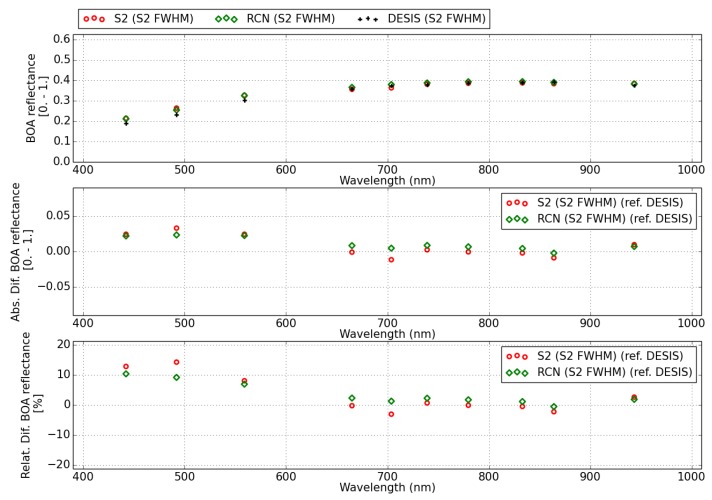
Surface reflectance for Sentinel-2B, DESIS and the Railroad Valley Playa RadCalNet (RCN) site of 28 June 2019. The spectra are convolved with Sentinel-2 spectral response function and the absolute and relative differences (middle, bottom plots) are calculated with respect to the DESIS spectrum.

For comparison, the RadCalNet and DESIS spectra have been convolved with Sentinel-2B spectral response functions. Figure 8 shows the surface reflectance spectra results for DESIS (black crosses), RadCalNet (labeled RCN, green diamonds) and Sentinel-2B (labeled S2, red circles). The upper plot shows the three spectra (DESIS, RadCalNet and Sentinel-2) convolved with Sentinel-2 B spectral response functions. The other two plots show the absolute (center) and relative (bottom) difference of Sentinel-2 (red circles) and RadCalNet (green diamonds) with respect to DESIS spectra.

The relative difference in surface reflectance between RadCalNet and DESIS is below 5% for wavelengths above 600 nm, while the relative difference increases up to 10% for shorter wavelengths (∼ 400–600 nm). This is consistent with DESIS results reported in [18], and it is explained as an overestimation of the AOT in remote sensing (AOT${}_{DESIS}$∼0.25) from arid sites with respect to the measured value (AOT${}_{RCN}$ = 0.035). However, comparisons with Sentinel-2 data (<30 min. overpass time difference) show a discrepancy. Sentinel-2 surface reflectance shows a difference of ∼12% with DESIS, which makes it consistent with RadCalNet, despite of the lack of DDV pixels (≤2%) in a Sentinel-2 scene and the resulting overestimation of the AOT (AOT${}_{S2}$ = 0.34).

The water vapor column retrieved for Sentinel-2 and DESIS uses the same algorithm but two different water vapor absorption regions for each of the sensors: 820 nm for DESIS and 940 nm for Sentinel-2. The water vapor retrieved from DESIS (WV${}_{DESIS}$ = 0.75 cm) and Sentinel-2B (WV${}_{S2}$ = 0.90 cm) is compared with the one reported by the RadCalNet site (WV${}_{RCN}$ = 0.72 cm). The difference between Sentinel-2 water vapor value and RadCalNet could be partially due to the time difference. RadCalNet reports a WV${}_{RCN}$ = 0.81 cm at 18:30 UTC, which yields a difference of ∼12% between Sentinel-2 and RadCalNet measurements. When compared to DESIS, Sentinel-2B shows a close agreement in the surface reflectance retrieved for wavelengths >600 nm.

## 6. Discussion

From a software engineering point of view, there are several design choices and improvements that will be considered in the future development of PACO. A necessary change is the transition of the current Python 2.7 code to Python 3.x as support for the former ends beginning of the year 2020. Furthermore, it is planned to implement the monochromatic and atmospheric lookup tables in a self-describing data format, which will feature convenience functionality like implicit file content metadata representation (describing the exact structure of the look-up tables) and file compression (e.g., HDF5, NetCDF, etc.).

The results of PACO performance for the estimation of atmospheric parameters (Table 3) can not be easily extrapolated to any other remote sensing data set since they are dependent on the distribution of reference values used in this study. Based on this study, it is recommended to characterize the performance as a function of the reference value, especially in the case of the AOT. Assuming that within each reference bin in Figure 4 the distribution of AOT or WV values is normal, we could approximate the uncertainty to 1σ gaussian error and conclude that 68% of scenes with values contained within each bin range would have an error equal to the bin uncertainty. However, this assumption is far from being completely correct, especially in the case of AOT, where some bins (AOT > 0.3) have not enough samples for a good uncertainty calculation. In addition, the amount of AERONET stations located in Earth sites, where the aerosol content is high, is rather low. It has been rather complicated to find the few statistics (in space and time) used for this validation study. In addition, each bin of AOT contains a non-uniform distribution in the aerosol particle characteristics, which might bias our results.

These points present three problems at the time of extrapolating the validation results for Earth’s monitoring missions: firstly, the geographical sampling itself is far from being uniform, so the statistical results are not representative of global monitoring statistics. Secondly, the AOT is calculated assuming the MODTRAN so called “rural” (Continental) aerosol model, which might not be completely true for all the randomly selected scenes. The last one concerns the blue/red ratio assumed in the DDV algorithms (currently ratio = 0.5 [31]), which is not constant over the globe [39].

Difference in AOT retrieval in Railroad Valley Playa and Gobabeb (due to the absence of DDV pixels) might affect the reflectance accuracy according to Equation (3). In order to minimize the effect of the uncertainty of the scene AOT calculation, and its effect on the accuracy of the surface reflectance, the AOT value measured on the RadCalNet sites at the time of the BOA measurements might be used as input for PACO’s atmospheric correction algorithms (for validation purposes). New software possibilities, like AOT input (already explored in [18]), are handy to compare the surface reflectance from different sensors from these types of homogeneous but arid sites, where DDV-based AOT extraction algorithms can not be applied or they are under low retrieved statistics. The results presented in this study are consistent with the same L2A validation study on the commissioning data of DESIS hyperspectral [18] camera system. Similar results were found when comparing DESIS surface reflectance results of scenes over the RadCalNet stations Gobabeb and Railroad Valley Playa [18].

## 7. Conclusions

PACO is a new atmospheric correction software. It is coded in Python and it is based on the well-known ATCOR IDL code. The migration from a proprietary programming language to Python allows the usage of the software as a third party module, making it very easy to be used in any remote sensing Ground Segment. It only requires the development of a module with the data model of the sensor input data. In addition, it takes the advantage of a globally maintained set of available Python libraries to perform the atmospheric correction of remote sensing satellite data.

A first release of the PACO software (under DLR licence) (Contact person: corresponding author) is currently operational for the multi-spectral sensors Sentinel-2 and Landsat-8 data and for the hyperspectral sensor DESIS as an L2A processor. The validation of multi-spectral sensors Sentinel-2 and Landsat-8 shows a retrieval of atmospheric parameters like the aerosol optical thickness and water vapor with an uncertainty of ∼30% and ∼10%, respectively. This uncertainty is dominated by an accuracy bias for very low values of AOT and WV and for high values of AOT. Therefore, the above-mentioned relative uncertainties are consistent with the precision for AOT values between 0.1 and 0.7, and WV values between 0.5 and 5 cm.

The validation results, when comparing Sentinel-2 surface reflectance retrieved with PACO and ground measurements at RadCalNet sites, yield an uncertainty consistent with MODIS limits for the test sites with enough DDV pixels. For arid sites, the uncertainty can be larger, especially at blue wavelengths (∼ 500 nm) due to the lack of DDV pixels to estimate the aerosol load in the atmosphere accurately.

Future releases of the software will allow the PACO user to obtain L2A products of other sensors, as the hyperspectral DESIS [18] and EnMAP [19]. The usage of the same software for the performance of the atmospheric correction will minimize the differences between the L2A products due to the software uncertainties.

## Figures and Tables

**Table 1:** Intermediate and final products resulting from PACO software execution.

Product Name	Label	Units	Number of Bands
Masks	hcw		1
Dense-dark Vegetation mask	ddv		1
Visibility index	visindex		1
Aerosol Optical Thickness	aot	1000 * [unit-less]	1
Water vapor column	wv	1000 * [cm]	1
Illumination	ilu		1
Quicklook	ql		3
BOA surface reflectance	atm	100 * [%]	number of bands (Table 2)

**Table 2:** BOA reflectance bands for Sentinel-2 and Landsat-8. The band name given by PACO is listed together with the mission original name and the proximate central wavelength (between brakets).

	PACO Band Name (Mission ∼ Central Wavelength (nm))		
**Bands**	**Sentinel-2 [35]**		**Landsat-8 [36]**
**(Band Number)**	**(Pixel ∼20 m)**	**(Pixel ∼10 m)**	**(Pixel ∼30 m)**
1	B1 (bands 1 ∼ 442)	B1 (bands 2 ∼ 492)	coastal (band 1, coastal aerosol ∼ 443)
2	B2 (bands 2 ∼ 492)	B2 (bands 3 ∼ 560)	blue (band 2, blue ∼ 482)
3	B3 (bands 3 ∼ 560)	B3 (bands 4 ∼ 665)	green (band 3, green ∼ 561)
4	B4 (bands 4 ∼ 665)	B8 (bands 8 ∼ 833)	red (band 4, red ∼ 654)
5	B5 (bands 5 ∼ 704)		nir (band 5, nir ∼ 864)
6	B6 (bands 6 ∼ 740)		cirrus (band 9, cirrus ∼ 1373)
7	B7 (bands 7 ∼ 780)		swir1 (band 6, swir ∼ 1609)
8	B8 (bands 8 ∼ 833)		swir2 (band 7, swir ∼ 2201)
9	B8a (bands 8a ∼ 864)		
10	B9 (bands 9 ∼ 945)		
11	B10 (bands 10 ∼ 1375)		
12	B11 (bands 11 ∼ 1612)		
13	B12 (bands 12 ∼ 2200)		

**Table 3:** Distribution results (A, P, and U) for the aerosol optical thickness (AOT) and water vapor column (WV) for Sentinel-2 and Landsat-8 with respect to AERONET data. The number of scenes (N) and the mean and median value of each distribution have been included.

	AOT${}_{550}$				WV [cm]			
**Satellite**	**N**	**Mean/Median**	**A ± P**	**U**	**N**	**Mean/Median**	**A ± P**	B
Sentinel-2	177	0.20/0.12	0.02 ± 0.12	0.12	375	1.8/1.5	0.06 ± 0.17	0.18
Landsat-8	51	0.15/0.09	0.02 ± 0.13	0.13	–	—	—	—

**Table 4:** RadcalNET sites with their corresponding extended area and surface reflectance variability (uniformity) across site considered for this study [47,48,49].

RadcalNet Site	Coordinates (${}^{o}$)		Area	ρ Site Variability
Name	Lon	Lat	(km × km)	(%)
La Crau (LCFR)	43.559	4.864	0.5	5
Gobabeb (GONA)	15.120	−23.600	0.5	3
Railroad Valley (RVUS)	38.497	−115.690	1.0	1.5

**Table 5:** DESIS and Sentinel-2 data acquisitions over the Railroad Valley Playa RadCalNet site.

Sensor	Tile	Time	Sun Zenith	Off-Nadir
		(UTC)	(deg)	(deg)
Sentinel-2 (B)	T11SPC	18:29:29	20	9
DESIS	3	18:53:01	19	3

## Abbreviations

The following abbreviations are used in this manuscript:

AERONET	AErosol RObotic NETwork
AOT	Aerosol Optical Thickness
APDA	Atmospheric Pre-corrected Differential Absorption
BOA	Bottom-Of-Atmosphere
BRD	Bi-directional Reflection Distribution
DDV	Dark Dense Vegetation
DEM	Digital Elevation Model
DESIS	DLR Earth Sensing Imaging Spectrometers
EnMAP	Environmental Mapping and Analysis Program
EO	Earth Observation
ESA	European Space Agency
IDL	Interactive Data Language
JSON	JavaScript Object Notation
LUT	Look-Up-Table
MBE	Mean Bias Error
MODIS	Moderate Resolution Imaging Spectroradiometer
MODTRAN	MODerate resolution atmospheric TRANsmission
PACO	Python-based Atmospheric COrrection
RadCalNet / RCN	Radiometric Calibration Network
RMSE	Root Mean Square Error
S2	Sentinel-2
TOA	Top-Of-Atmosphere
VNIR	Visible and Near-InfraRed
SWIR	Short Wavelength InfraRed
WV	Water Vapor

## References

[1] Vermote E.F., El Saleous N., Justice C.O., Kaufman Y.J., Privette J.L., Remer L., Roger J.C., Tanré D. (1997). Atmospheric correction of visible to middle-infrared EOS-MODIS data over land surfaces: Background, operational algorithm and validation. J. Geophys. Res. Atmos..

[2] Thompson D., Guanter L., Berk A., Gao B.C., Richter R., Schläpfer D., Thome K. (2018). Retrieval of Atmospheric Parameters and Surface Reflectance from Visible and Shortwave Infrared Imaging Spectroscopy Data. Surv. Geophys..

[3] Franch B., Vermote E., Roger J.C., Murphy E., Becker-Reshef I., Justice C., Claverie M., Nagol J., Csiszar I., Meyer D. (2017). A 30+ Year AVHRR Land Surface Reflectance Climate Data Record and Its Application to Wheat Yield Monitoring. Remote Sens..

[4] Ientilucci E.J., Adler-Golden S. (2019). Atmospheric Compensation of Hyperspectral Data: An Overview and Review of In-Scene and Physics-Based Approaches. IEEE Geosci. Remote Sens. Mag..

[5] Bernstein L.S., Adler-Golden S.M., Jin X., Gregor B., Sundberg R.L. Quick atmospheric correction (QUAC) code for VNIR-SWIR spectral imagery: Algorithm details. Proceedings of the 2012 4th Workshop on Hyperspectral Image and Signal Processing: Evolution in Remote Sensing (WHISPERS).

[6] Gao B.C., Montes M.J., Ahmad Z., Davis C.O. (2000). Atmospheric correction algorithm for hyperspectral remote sensing of ocean color from space. Appl. Opt..

[7] Miller C.J., Shen S.S., Lewis P.E. (2002). Performance assessment of ACORN atmospheric correction algorithm. Algorithms and Technologies for Multispectral, Hyperspectral, and Ultraspectral Imagery VIII.

[8] Qu Z., Kindel B.C., Goetz A.F.H. (2003). The high accuracy atmospheric correction for hyperspectral data (hatch) model. IEEE Trans. Geosci. Remote Sens..

[9] Richter R. (1998). Correction of satellite imagery over mountainous terrain. Appl. Opt..

[10] Vermote E.F., Vermeulen A. ATMOSPHERIC CORRECTION ALGORITHM: SPECTRAL REFLECTANCES (MOD09). https://modis.gsfc.nasa.gov/data/atbd/atbd_mod08.pdf.

[11] Louis J., Pflug B., Main-Knorn M., Debaecker V., Mueller-Wilm U., Iannone R.Q., Giuseppe Cadau E., Boccia V., Gascon F. Sentinel-2 Global Surface Reflectance Level-2a Product Generated with Sen2Cor. Proceedings of the IGARSS 2019-2019 IEEE International Geoscience and Remote Sensing Symposium.

[12] Hagolle O., Huc M., Pascual D., Dedieu G. (2015). A Multi-Temporal and Multi-Spectral Method to Estimate Aerosol Optical Thickness over Land, for the Atmospheric Correction of FormoSat-2, LandSat, VENS and Sentinel-2 Images. Remote Sens..

[13] Lonjou V., Desjardins C., Hagolle O., Petrucci B., Tremas T., Dejus M., Makarau A., Auer S., Comerón A., Kassianov E.I., Schäfer K. (2016). MACCS-ATCOR joint algorithm (MAJA). SPIE Remote Sensing.

[14] Doxani G., Vermote E., Roger J.C., Gascon F., Adriaensen S., Frantz D., Hagolle O., Hollstein A., Kirches G., Li F. (2018). Atmospheric Correction Inter-Comparison Exercise. Remote Sens..

[15] Flood N., Danaher T., Gill T., Gillingham S. (2013). An Operational Scheme for Deriving Standardised Surface Reflectance from Landsat TM/ETM plus and SPOT HRG Imagery for Eastern Australia. Remote Sens..

[16] Claverie M., Ju J., Masek J.G., Dungan J.L., Vermote E.F., Roger J.C., Skakun S.V., Justice C. (2018). The Harmonized Landsat and Sentinel-2 surface reflectance data set. Remote Sens. Environ..

[17] Berdou G., Shrestha S., Hahn M. (2019). Integration of sentinel-2 and landsat-8 data for surface reflectance time-series analysis. ISPRS-Int. Arch. Photogramm. Remote Sens. Spat. Inf. Sci..

[18] Alonso K., Bachmann M., Burch K., Carmona E., Cerra D., de los Reyes R., Dietrich D., Heiden U., Hölderlin A., Ickes J. (2019). Data Products, Quality and Validation of the DLR Earth Sensing Imaging Spectrometer (DESIS). Sensors.

[19] Guanter L., Kaufmann H., Segl K., Foerster S., Rogass C., Chabrillat S., Kuester T., Hollstein A., Rossner G., Chlebek C. (2015). The EnMAP spaceborne imaging spectroscopy mission for earth observation. Remote Sens..

[20] Richter R., Schläpfer D., Müller A. (2006). An automatic atmospheric correction algorithm for visible/NIR imagery. Int. J. Remote Sens..

[21] Richter R., Schlapfer D., Müller A. (2011). Operational Atmospheric Correction for Imaging Spectrometers Accounting for the Smile Effect. IEEE Trans. Geosci. Remote Sens..

[22] Richter R., Wang X., Bachmann M., Schläpfer D. (2011). Correction of cirrus effects in Sentinel-2 type of imagery. Int. J. Remote Sens..

[23] Richter R., Heege T., Kiselev V., Schläpfer D. (2014). Correction of ozone influence on TOA radiance. Int. J. Remote Sens..

[24] Atmospheric/Topographic Correction for Airborne Imagery. https://www.rese-apps.com/pdf/atcor4_manual.pdf.

[25] Berk A., Anderson G.P., Acharya P.K., Shettle E.P. (2008). MODTRAN 5.2.0 User’s Manual.

[26] Fontenla J.M., Harder J., Livingston W., Snow M., Woods T. (2011). High-resolution solar spectral irradiance from extreme ultraviolet to far infrared. J. Geophys. Res. Atmos..

[27] Thuillier G., Hersé M., Labs D., Foujols T., Peetermans W., Gillotay D., Simon P., Mandel H. (2003). The Solar Spectral Irradiance from 200 to 2400 nm as Measured by the SOLSPEC Spectrometer from the Atlas and Eureca Missions. Sol. Phys..

[28] Platnick S.E.A. (2017). MODIS Atmosphere L3 Eight-Day Product.

[29] Wan Z., Hook S.H.G. (2018). MYD11A2 MODIS/Aqua Land Surface Temperature/Emissivity 8-Day L3 Global 1km SIN Grid V006.

[30] Atmospheric and Topographic Correction (ATCOR Theoretical Background Document). https://www.rese-apps.com/pdf/atcor_ATBD.pdf.

[31] Kaufman Y.J., Wald A.E., Remer L., Gao B.C., Li R.R., Flynn L. (1997). The MODIS 2.1-*μ*m channel-correlation with visible reflectance for use in remote sensing of aerosol. IEEE Trans. Geosci. Remote Sens..

[32] Schläpfer D., Borel C.C., Keller J., Itten K.I. (1998). Atmospheric Precorrected Differential Absorption Technique to Retrieve Columnar Water Vapor. Remote Sens. Environ..

[33] Richter R. (1990). A fast atmospheric correction algorithm applied to Landsat TM images. Int. J. Remote Sens..

[34] Richter R., Bachmann M., Dorigo W., Muller A. (2006). Influence of the Adjacency Effect on Ground Reflectance Measurements. IEEE Geosci. Remote Sens. Lett..

[35] Drusch M., Bello U.D., Carlier S., Colin O., Fernandez V., Gascon F., Hoersch B., Isola C., Laberinti P., Martimort P. (2012). Sentinel-2: ESA’s Optical High-Resolution Mission for GMES Operational Services. Remote Sens. Environ..

[36] Barsi J., Lee K., Kvaran G., Markham B., Pedelty J. (2014). The Spectral Response of the Landsat-8 Operational Land Imager. Remote Sens..

[37] Holben B., Eck T., Slutsker I., Tanré D., Buis J., Setzer A., Vermote E., Reagan J., Kaufman Y., Nakajima T. (1998). AERONET—A Federated Instrument Network and Data Archive for Aerosol Characterization. Remote Sens. Environ..

[38] Makarau A., Richter R., Schläpfer D., Reinartz P. (2016). APDA Water Vapor Retrieval Validation for Sentinel-2 Imagery. IEEE Geosci. Remote Sens. Lett..

[39] Vermote E.F., Kotchenova S. (2008). Atmospheric correction for the monitoring of land surfaces. J. Geophys. Res. Atmos..

[40] Remer L.A., Kaufman Y.J., Tanré D., Mattoo S., Chu D.A., Martins J.V., Li R.R., Ichoku C., Levy R.C., Kleidman R.G. (2005). The MODIS Aerosol Algorithm, Products, and Validation. J. Atmos. Sci..

[41] S2 MPC, Level 2A Data Quality Report. https://sentinel.esa.int/documents/247904/685211/Sentinel-2-L2A-Data-Quality-Report.

[42] Pflug B., Main-Knorn M., Makarau A., Richter R. (2015). Validation of aerosol estimation in atmospheric correction algorithm ATCOR. ISPRS-Int. Arch. Photogramm. Remote Sens. Spat. Inf. Sci..

[43] Obregón M.A., Rodrigues G., Costa M.J., Potes M., Silva A.M. (2019). Validation of ESA Sentinel-2 L2A Aerosol Optical Thickness and Columnar Water Vapour during 2017-2018. Remote Sens..

[44] RadCalNet Quick Start Guide. https://www.radcalnet.org/resources/RadCalNetQuickstartGuide_20180702.pdf.

[45] Bouvet M., Thome K., Berthelot B., Bialek A., Czapla-Myers J., Fox N.P., Goryl P., Henry P., Ma L., Marcq S. (2019). RadCalNet: A Radiometric Calibration Network for Earth Observing Imagers Operating in the Visible to Shortwave Infrared Spectral Range. Remote Sens..

[46] CEOS Reference: QA4EO-WGCV-IVO-CSP-002-BTCN. https://www.radcalnet.org/sites/BTCN/documentation/Site%20documentation/QA4EO-WGCV-IVO-CSP-002_BTCN_20180405.pdf.

[47] CEOS Reference: QA4EO-WGCV-IVO-CSP-002-LCFR. https://www.radcalnet.org/sites/LCFR/documentation/Site%20documentation/QA4EO-WGCV-IVO-CSP-002_LCFR_20180405.pdf.

[48] CEOS Reference: QA4EO-WGCV-IVO-CSP-002-RVUS. https://www.radcalnet.org/sites/RVUS/documentation/Site%20documentation/QA4EO-WGCV-IVO-CSP-002_RVUS_20180404.pdf.

[49] CEOS Reference: QA4EO-WGCV-IVO-CSP-002-GONA. https://www.radcalnet.org/sites/GONA/documentation/Site%20documentation/QA4EO-WGCV-IVO-CSP-002_GONA_20180405.pdf.

